# Integrating health disparities and environmental health into community-based medical education: a qualitative study

**DOI:** 10.1186/s12909-025-08485-w

**Published:** 2026-01-27

**Authors:** Nazli Hossain, Sonia Ijaz Haider, Tayyeba Anbreen, Faiza Siddiqui

**Affiliations:** 1 Department of Obstetrics and Gynecology, Dow University of Health Sciences, Karachi, Pakistan; 2 Dow Institute of Health Professions Education, Dow University of Health Sciences, Karachi, Pakistan

**Keywords:** Community-based medical education, Health disparities, Environmental health, Medical curriculum, Qualitative study, Pakistan

## Abstract

**Background:**

Community-Based Medical Education (CBME) provides experiential learning that links medical training with real-world health needs. Integrating themes of health disparities and environmental health within CBME is critical for developing socially accountable physicians in low- and middle-income countries. However, limited evidence from Pakistan describes effective approaches for embedding these domains in undergraduate curricula. This study explored stakeholders perceptions on pragmatic approaches for incorporating health disparity and environmental health within competency-based learning at a public medical university.

**Methods:**

A qualitative study using focus group discussions (FGDs) was conducted at Dow University of Health Sciences (DUHS), Karachi, Pakistan, f

rom April to June, 2025. Two FGDs were held with final-year medical students (*n* = 18; 9 participants per group), three with residents from different clinical departments (*n* = 30; 10 per group), and one with faculty members (*n* = 12). Participants were selected through convenience (learners) and purposive (faculty) sampling. The discussions were audio-recorded and transcribed verbatim, and Braun and Clarke's six steps approach was applied for thematic analysis.

**Results:**

Six themes emerged: (1) Enhancing medical education through CBME, (2) Addressing health inequalities through community engagement, (3) Issues in implementation (logistics, safety, cultural barriers), (4) Creating sustainable long-term partnerships with communities, (5) Integration of environmental and public health education, and (6) Monitoring and Evaluation for sustainable CBME. Participants emphasized putting early integrated longitudinal placements, institutional support, and collaboration with local organizations into practice to promote equitable and environmentally responsible practice.

**Conclusions:**

Health-disparity and environmental health content integrated within CBME will improve learning context and responsiveness to the community. Structured planning, faculty training, and stakeholder partnerships are essential for sustainable implementation and for preparing graduates to address emerging community health challenges.

**Supplementary Information:**

The online version contains supplementary material available at 10.1186/s12909-025-08485-w.

## Introduction

Community based education is an educational approach in which students are provided opportunities to engage within the community. Such contextual learning opportunities facilitate the students to learn meaningfully, develop critical and problem solving skills [1]. This exposure also helps the students to comprehend the influence of social determinants on patients, their family and the community.

Community based teaching about health disparities enables learners to gain a comprehensive understanding of the social contextual factors that contribute to the disparities through instruction about disparities in the community settings [2]. This instills a sense of responsibility and a desire to advocate for the needs of the people and communities, who lack the resources to do so. In one study, students reflected that this experience taught them that the place, environment and conditions in which people live influence their ability to make healthy choices and get access to care [3]. In most of the studies, this engagement with the community is perceived as giving back to the community, particularly when the practical projects make a positive difference in the lives of people [4]. In one study, first year medical students were taught health equity and community health using hybrid and virtual experiential learning opportunities which demonstrated increased knowledge and interest in working with medically underserved communities [5]. Evidence indicates that CBME played an important role in the delivery of undergraduate medical education in rural communities, where community could benefit more from the initiatives of promoting health and preventing illness than from therapeutic services [6]. In undergraduate medical education, CBME has been embedded as both short and long term longitudinal student clerkships. For short term clerkships, while students demonstrate enthusiasm and motivation to contribute to community, for long placements they develop a sense of companionship within the community, particularly with their preceptors [7].

Teaching about climate change or environmental health and consciousness has also gained momentum in medical education since WHO estimated that 23% of global diseases are attributed to environmental factors [8]. The aim is to prepare future healthcare force to address health issues related to climate change, pollution, and lead contamination within the communities [9]. In one study, the impact of integrating environmental health into medical school curricula was explored. After six weeks completion of the module, first year medical students reported increased awareness about environmental issues and strategies for addressing them [10]. In another study, second year medical students were taught virtually about environmental topics in medical education. The study reported increase in awareness and knowledge, however no change was reported in deep personal attitude [11]. In another study, the content for teaching climate change was discussed with the leadership and the faculty of a medical school. Findings indicated the importance of developing climate specific resources which fit with the vision of the medical school, and the cultural context [12]. Further evidence indicates that teaching environmental consciousness through CBME has the potential benefit of fostering skills of problem solving, negotiation, leadership, and teamwork among students.

In Pakistan, inclusion of community based medical education in undergraduate medical education is incorporated in the medical education as per requirement of the Pakistan Medical and Dental Council [13]. However, evidence exploring implementation of community based education is limited. There is need to explore the way different topics are taught to the students, their experiences of engaging with the community and address any challenges which students face during placements in the community. This will be useful in ensuring that learning is maximized for the students and the communities also benefit from student engagement. Most of the existing evidence is focused on the need for implementing community based medical education in the curriculum [14]. There is little empirical evidence which explores students perceptions or experiences related to its implementation. An earlier study explored satisfaction of knowledge and skills community orientated skills in medical undergraduates at Karachi. Compared to basic and clinical sciences, students perceived the community orientated curricula was unable to meet the required standards [15]. In another study which explored perceptions of undergraduate medical students at ophthalmic medical camps as part of their community based medical education reported increase in knowledge, and improved communication skills regarding taking focused history and counseling patients [16]. Furthermore there is scant evidence which determines the efforts to integrate topics such as health disparities, public health engagement and environmental consciousness in the community based medical education in Pakistan.

Building on this background, the present study explores multi-stakeholder perspectives of students, residents, and faculty on feasible strategies for integrating health disparity and environmental health domains within community-based medical education at a Public Medical University, in Karachi, Pakistan. The study address the following research question; How do learners and faculty perceive feasible strategies to integrate health disparities and environmental health into CBME at Dow University of Health Sciences, Karachi, in Pakistan?

## Methods

A total of six focus group discussions (FGDs) were conducted between April to June 2025 at Dow University of Health Sciences (DUHS), Karachi, Pakistan. Two FGDs were held with final-year medical students (*n* = 18; 9 per group), three with residents from different clinical departments (*n* = 30; 10 per group), and one with faculty members (*n* = 12). Each group size was within the recommended qualitative range (8–12 participants) [17]. Students and residents participated in separate FGDs to prevent hierarchical or power-related influences that might constrain open discussion.

For learners (students and residents), a convenience sampling approach was used based on their availability and willingness to participate. Meanwhile, faculty members were purposively sampled, recruiting individuals with a minimum of five years of teaching experience and representing both basic and clinical sciences. Recruitment was carried out through departmental circulars, email invitations, and personal contact. Out of 71 invitations sent out, 60 accepted while an overall participation rate of 85% was reached. Non-participation was owing to scheduling clashes and examination commitments. This multi-stakeholder design ultimately ensured diversity of views across learners and educator cohorts while maintaining feasible and qualitative depth.

### Data collection

The research team consisted of three female faculty members from the Dow University of Health Sciences (DUHS) with training in the fields of medical education and public health. Although all researchers were affiliated with DUHS, none had a supervisory or evaluative role concerning the student or resident participants. The authors acknowledged the possibility that institutional hierarchy and professional familiarity influenced participants' responses.

To ensure reflectivity and minimize bias, the moderators maintained neutrality on confidentiality, voluntary participation, and differing opinions of participants. Moreover after each FGD, peer debriefing sessions were held to reflect on personal assumptions and maintain reflexive awareness. To reduce individual bias, all the analytical decisions were discussed collectively by the group members. An audit trail was also kept to document decisions and ensure transparency.

Multiple strategies were used to enhance the credibility of the findings. Authors engaged in prolonged focus group discussions to gain in-depth understanding of the perspectives of participants. This was followed by member checking among all the authors to confirm the accuracy of the views of all the participants, and using triangulation to validate the findings. Dependability was ensured by keeping an audit trail of all decisions, coding revisions, and analytical notes. Confirmability was carried out by the reflexive journal which was kept by the researchers. In addition, two researchers independently coded the data and met to resolve any differences. Transferability was achieved by providing contextual details about both the institution and the participants. Further verbatim quotes are included to facilitate the readers to draw relevance to their own settings. All the FGDs were held in a quite seminar room within the university ensuring both accessibility and privacy to the participants. The audience comprised only participants, moderators, and note-taker. Audio-recording was done with written and verbal consent from all participants, together with field notes taken at the time of the demonstration. The participants were assured of anonymity and confidentiality, and identifiers were later removed from the transcripts.

The discussions were mainly in English and Urdu, allowing the participants to switch languages when they felt clarity was needed. Transcripts were prepared verbatim and then translated into English by bilingual transcribers. For validation purposes, back-translation was done and 20% of the transcripts underwent cross-checking by an independent bilingual reviewer.

The semi-structured FGD guide (Annexure I) was developed from literature on community-based education, health disparities, and environmental health. It was pilot-tested with two students and one faculty member to assess clarity and sequencing. Minor wording and order adjustments were made after the first FGD (e.g., combining overlapping prompts on environmental health). The final guide included in Annexure I matches exactly the instrument used for all subsequent FGDs.

Data collection continued until thematic saturation was reached, as the point when no new codes or concepts emerged from the data [18]. Saturation was observed after the fifth FGD, at which time the team agreed that additional sessions would yield repetition rather than novel insights.

### Data analysis

Thematic analysis followed the six-step framework proposed by Braun and Clarke [19]. A hybrid inductive–deductive approach was used: while health disparities and environmental health were considered a-priori sensitizing concepts, additional codes and themes were derived inductively from participants’ narratives. All transcripts were anonymized and uploaded into NVivo 12 for systematic organization.

In an independent process, both researchers (SH and FS) carried out open initial coding on one student and one resident transcript, to identify the key findings. Through an iterative discussion, overlapping codes were merged and a working codebook, with definitions and exemplar quotations, was created [20]. This working codebook continued to be re-evaluated as the analysis of subsequent transcripts proceeded from there until saturation was reached. Final Structure consisted of six major themes and nineteen subthemes, along with coding trees (parent–child relationship) shown in the attached Supplement 2.

To maintain internal consistency in the analysis, an additional 20% of transcripts were double coded for both researchers with intercoder agreement of > 85%. Disagreements within this double coding group were then resolved through consensus discussions [21]. All coded data and final themes were reviewed for conceptual clarity by the senior author (NH).

Credibility was enhanced by member checking with six participants (two from each cadre) for thematic accuracy, peer debriefing within the research team, and exhaustive documentation of audit trails in NVivo of all coding and codebook revisions and analytic memos [22]. Consequently the analytic process was transparent, dependable, and confirmable.

Ethical Approval Ethical approval was obtained for this study from the Institutional Review Board of the Dow University of Health Sciences, Karachi (IRB No. 3978-DUHS; approval date April 2025). Before participation, each individual was given a written information sheet about the nature of the study, confidentiality of responses, and the voluntary nature of participation. Written informed consent was obtained for both participation and audio-recording, with verbal confirmation reiterated immediately before each focus-group session. Participants were informed that anonymized quotations might appear in publications and that they could withdraw at any point without penalty.

The audio files and transcripts were kept in a password-protected, encrypted institutional server which was accessible only by research team members. Transcription de-identification took place during transcription by removing all personal, departmental, or geographic identifiers, replacing them with assigned coded labels (e.g., S4 = Student 4, R7 = Resident 7, F2 = Faculty 2). Consent forms were kept separately locked in a cabinet. Data will be stored for five years and then securely destroyed thereafter.

All researchers were members of DUHS and engaged in academic roles related to CBME, but they had no roles involving direct supervision or assessment of any of the participants in this study. To balance against institutional or positional bias, neutral moderators facilitated the discussions, and participants were assured of confidentiality and voluntary participation. Also, the team held peer-debriefing sessions and reflexive memoing for critical reflection on assumptions. These measures, thus, ensured that findings would authentically mirror the perspectives of participants while maintaining ethical and analytic integrity.

## Results

Analysis of the six FGDs resulted in six overarching themes and nineteen sub-theme elucidating participants’ perceptions of integrating health-disparity and environmental-health content within community-based medical education (CBME). Table 1 presents the theme matrix with exemplar quotations labeled by stakeholder group.

**Table 1:** Themes, sub-themes, and exemplar quotations by stakeholder group

Theme	Sub-Theme	Exemplar Quotations (Labeled)
1. Enhancing Medical Education through CBME	•Bridging Theory and Practice • Early Community Exposure • Hands-On Learning	“We know the theory but not how to apply it in real communities.” (S4) “Exposure in second year would help link pre-clinical learning to real problems.” (R3) “Students learn most when they actually work with communities.” (F2)
2. Addressing Health Disparities through CBME	•Equitable Access to Care • Community Trust Building • Environmental Determinants of Health	“Patients from rural areas cannot reach hospitals easily.” (S7) “If we keep returning to the same sites, people start trusting us.” (R8) “Polluted water and waste piles increase disease—students should study these issues.” (F5)
3. Challenges in Implementing CBME	•Logistical Constraints • Safety Concerns • Institutional Barriers	“No transport or funds for community visits.” (R10) “Female students cannot travel alone to distant villages.” (S2) “Without institutional support, CBME remains fragmented.” (F4)
4. Integrating Environmental and Public-Health Education	•Environmental Awareness • Hygiene & Waste Management • Sustainable Practices	“Learning about pollution and waste taught us prevention, not just treatment.” (S3) “Each module should mention how it affects the environment.” (R6) “Green-clinic projects could teach sustainability in practice.” (F1)
5. Strengthening Community Engagement	•Long-Term Partnerships •Collaboration with Local Leaders •Mentorship Support	“Following up after a few months shows continuity and commitment.” (S6) “Community leaders help us communicate better.” (R9) “Faculty mentorship sustains these outreach efforts.” (F7)
6. Monitoring and Evaluation for Effective CBME	•Feedback and Recognition •Assessment of Impact	“Certificates or reflection sessions motivate participation.” (S5) “Collecting feedback from communities tells us if learning had an effect.” (R12) “Tracking outcomes keeps programs accountable.” (F6)

### Integration of health disparity and environmental dimensions

Participants consistently associated both the social and environmental determinants of health. Students and residents perceived that poor sanitation, waste disposal, and air pollution aggravated inequities in underserved areas which emphasizes the need to teach both dimensions simultaneously within CBME. Faculty accentuated designing modules where health disparities and environmental health interconnect such as community sanitation drives, water-testing projects, or climate-related health awareness sessions.

## Discussion

The findings of this study indicate that integrating health disparities and environmental health within community-based medical education (CBME) can strengthen socially accountable and ecologically responsive medical training. Each of the six themes have a unique but related impact on the design, implementation, and evaluation of the curriculum.

### Theme 1: Improving CBME through experiential learning

Participants stressed continued exposure to the community and participation with marginalized groups during the earlier years and throughout training [23]. The rationale for placing students longitudinally beginning in the pre-clinical years is to help them build their gradual understanding of clinical context and clinical reasoning processes. This connects with Kern's curriculum design framework contextual need assessment phase [24] and lends support to earlier proof of the fact that prolonged experiential learning enhances empathy, critical thinking, and social accountability.

### Theme 2: Equity in CBME interpretation for health disparities

Students and faculty alike realized that understanding social determinants of health and disparity in access to care was a major issue. Social accountability can be promoted through the incorporation of advocacy training, fieldwork in the underserved areas, and health equity modules into the curriculum. These are corresponding to the social accountability frame [25] and affirm that medical education needs to stay relevant concerning community needs [26]. For example, students could be assigned to identify community health issues like sanitation or malnutrition and propose realistic solutions that can then be presented to provincial health authorities for action.

### Theme 3: Hurdles in implementation

Participants of the study reported that they get exposure to the community, mostly in the tertiary care where there are limited patients. Therefore, they are unable to comprehend the social determinants of health and the challenges faced by the underserved population. Although medical colleges have community placement programs within their curriculum, however, they are constantly faced with challenges such as lack of defined learning outcomes, safety issues within the community, restricted funding and continuous follow-up for longer duration of the placement. Suggestions to overcome these challenges included offering CBME teaching workshops to enhance faculty competence, and coordination with community leaders to ensure safety protocols, consist follow-up and organize fundraising.

### Theme 4: Integrating environmental and public health

The findings of the study highlight that environmental consciousness or environmental health is taught, however, the topics are more theoretical, and their integration into practice is lacking. Participants suggested to address this by integrating it as a longitudinal theme by focusing on principles of WHO’s Planetary Health Education [27]. in the preclinical years and then environmental topics can be merged with teaching of clinical years in which eco-friendly hospital management, and green healthcare practices can promote sustainable professional behaviours [11]. Furthermore adopting initiatives such waste management drives, clean-water projects, encouraging the use of environmental friendly technologies, electronic documentation; and promoting an organizational culture of greener environment will transform from theory to practice [28, 29]

### Theme 5: Build on community engagement

Participants stressed the need to build partnerships and collaborations with local authorities, Non-Government Organizations, government agencies. Suggestions included to formalize student placement within the community by agreeing on memoranda of understanding with community organizations. This will facilitate in building trust and a shared sense of responsibility by both the students and the community members for improving their health outcomes [30]. In this way, it will be possible to address specific cultural, linguistic, and accessibility barriers through engaging community facilitators.

### Theme 6: Monitoring and evaluation for effective CBME

Participants strongly emphasized that after implementation of CBME is implemented, it’s imperative to monitor and evaluate it. This should be done regularly to ensure students are learning out of their community engagement, and the community is also benefiting from students engagement. Suggestions included to seek feedback from the community, collecting and analyzing data on disease prevalence, health behaviors, and community health indicators, [31], as well as assessment of students learning, their research outputs and capstone projects [32]. Participants proposed some indicators such as student reflections, community satisfaction, participation rates, and health-promotion outcomes for a structured monitoring and evaluation system (Supplements 5–6) [31, 32].

### Practice and policy implications

Collectively, the findings of the present study highlight the need for an integrated framework for CBME in medical curricula in Pakistan, which addresses both health equity and environmental sustainability simultaneously. Through implementation of these findings into supplementary materials-longitudinal CBME maps; learning outcomes; monitoring and evaluating (M&E) matrices; and assessment rubrics-institutions can apply ways of contextually grounded, evidence informed approaches to curriculum redesign (Supplements 1–6).

The M&E matrix proposed by this study (supplement 4) provides a systematic method for integrating health inequality and environmental health competences in national medical curricula. It corresponds directly to Pakistan’s Medical and Dental curriculum guidelines for undergraduate medical education (Pakistan Medical and Dental Council, 2024) and HEC's Quality Assurance Framework [33] as both emphasize outcome-based education with a community-engaged, sustainable approach. Besides operationalizing international standards-set for improvement purposes by the WFME Global Standards for Quality Improvement [34], the matrix provides measurable indicators taking inputs, activities, outputs, and outcomes that can support accreditation, institutional audits, and curriculum reviews.

From the policy level, this would-be framework enables medical universities along with the regulator to track the social and environmental responsiveness of their programs. Such frameworks also create accountability of programs to learners and communities. It provides a replicable model for adoption by other health-professions institutions interested in institutionalizing Community-Based Medical Education (CBME) through evidence-informed and context-sensitive indicators. The integration of this matrix into national quality assurance systems would facilitate in developing future doctors who in addition to being clinically competent, are also socially responsible and environmentally conscious citizens.

### Limitations

There are various limitations pertaining to this study, and the limitations should be considered while interpreting the study findings. One limitation is that it was conducted in one institution in an urban context and may not be able to transfer the results to rural or private-sector institutions in which community dynamics differ. Second, while we had focus groups composed of 8–12 participants to include a variety of voices, perhaps larger groups introduced some level of restraint on some individuals to present their thoughts with depth. Third, while we ensured participants that their responses would be confidential, affiliation of the researchers might have led them to give more favorable responses toward the institution. We tried to alleviate this risk by engaging neutral moderators who were not in any supervisory role and stressed voluntary, anonymous participation.

Besides, since community members were not part of the current study, and document analysis or field observations for triangulation of data were never carried out, there exists the limitation of an understanding. There is a need for future research that can incorporate stakeholder communities and apply mixed-method triangulation to strengthen contextual validity. Lastly, environmental health was not so prominently discussed by the participants after an extensive debate on health inequities, possibly according to the current curricular orientation. This accentuates the glaring gap to integrate environmental health concepts into community-oriented programs in medical education. Notwithstanding these limitations, this study does significantly contribute to understanding multi-stakeholder perspectives of embedding health equity and environmental sustainability into undergraduate medical education while developing a workable framework for settings of a similar nature.

## Conclusion

In a developing country, Pakistan finds the eradication of health inequities and the promotion of environmental awareness as burning issues that can further support community-based medical education. To begin, community-based medical education in undergraduate programs across the country needs to be planned and implemented in all medical colleges. Health priorities in the underserved communities should be linked to complementing short-and long-term placements that enhance students' learning experiences while addressing community concerns. Training of faculty and partnerships with government, NGOs, and industry should be built around issues of security, risk, logistics, and accessibility to the community. After continuous monitoring and evaluation, interventions should focus on addressing contextual issues surrounding health inequities, in relation to bringing about an understanding of relative contributions toward environmental health, via community-based medical education.

## Supplementary Information

Supplementary Material 1.

Supplementary Material 2.

## Data Availability

All data generated or analysed during this study are included in this published article.
