# Supplementary material for: Integrating health disparities and environmental health into community-based medical education: a qualitative study
Source: BMC Med Educ. 2026 Jan 27;26:311. doi: 10.1186/s12909-025-08485-w (PMC12918272; doi:10.1186/s12909-025-08485-w)
Supplement: Supplementary file 1 — Supplementary Material 1. [file 12909_2025_8485_MOESM1_ESM.docx]

**Supplement 1. COREQ Checklist**

| **Domain / Item** | **Description** | **Page / Section in Manuscript** |
| --- | --- | --- |
| **Domain 1 – Research Team & Reflexivity** |  |  |
| Personal characteristics | Female faculty trained in medical education & public health | Methods – Data Collection (p. 4) |
| Credentials | MBBS, MPH, MHPE | Methods (p. 4) |
| Occupation | Faculty at DUHS (no supervisory role over participants) | Methods (p. 4) |
| Experience / training | Experienced qualitative researchers | Methods (p. 4) |
| Relationship established | Prior institutional familiarity only | Methods (p. 4) |
| Reflexivity measures | Peer debriefing & reflexive journaling | Methods (p. 4-5) |
| **Domain 2 – Study Design** |  |  |
| Theoretical framework | Braun & Clarke (2006) six-step thematic analysis | Analysis (p. 6) |
| Participant selection | Convenience (students/residents); purposive (faculty) | Sampling (p. 3–4) |
| Setting | DUHS seminar room (neutral/private) | Data Collection (p. 4) |
| Sample size | 6 FGDs (18 students, 30 residents, 12 faculty) | Sampling (p. 3) |
| Non-participation | 71 invited → 60 participated (85 %) | Sampling (p. 4) |
| Data collection method | Semi-structured FGD guide (pilot-tested) | Data Collection (p. 5) |
| Ethical clearance | IRB Approval | Ethical clearance IRB No. 3978-DUHS (p. 6–7) |
| Recording / duration | Audio + field notes; 60–75 min | Data Collection (p. 5) |
| Data saturation | Reached after 6th FGD | Data Collection (p. 5) |
| **Domain 3 – Analysis & Findings** |  |  |
| Number of coders | Two independent researchers | Analysis (p. 6) |
| Coding tree | Provided in Supplement |  |
| Derivation of themes | Hybrid (inductive + deductive) | Analysis (p. 6) |
| Software | NVivo 12 | Analysis (p. 6) |
| Participant checking | Member-checking with 6 participants | Methods (p. 6) |
| Quotations | Labelled (S#, R#, F#) | Table 1 |
| Clarity of themes | Six themes / 19 sub-themes | Results + Table 1 |
| Reflexivity | Explicitly described | Methods (p. 4-5) |

**Supplement 2. Coding Tree / Codebook Excerpt**

| **Parent Theme** | **Sub-Theme** | **Definition** | **Sample Code** | **Representative Quote** |
| --- | --- | --- | --- | --- |
| Enhancing Medical Education through CBME | Early Community Exposure | Opportunities for early practice engagement | early exposure | “Exposure in second year would help link pre-clinical learning to real problems.” (R3) |
|  | Bridging Theory & Practice | Applying classroom learning to community contexts | apply theory | “We know the theory but not how to apply it in real communities.” (S4) |
| Addressing Health Disparities | Community Trust Building | Sustained presence and relationship with community members | trust building | “If we keep returning … people start trusting us.” (R8) |
|  | Equitable Access to Care | Recognizing systemic barriers to care | access issues | “Patients from rural areas cannot reach hospitals easily.” (S7) |
| Implementation Challenges | Safety Concerns | Travel and gender barriers | safety issues | “Female students cannot travel alone to distant villages.” (S2) |
|  | Logistical Constraints | Transport / funding limitations | logistics | “No transport or funds for community visits.” (R10) |
| Environmental & Public Health | Hygiene & Waste Management | Understanding environmental determinants | waste management | “Learning about pollution and waste taught us prevention.” (S3) |
|  | Sustainable Practices | Promoting eco-friendly approaches to care | green initiatives | “Green-clinic projects could teach sustainability.” (F1) |
| Strengthening Community Engagement | Long-Term Partnerships | Maintaining continuity with communities | follow-up | “Following up after a few months shows commitment.” (S6) |
| Monitoring & Evaluation | Feedback and Recognition | Reflective learning and acknowledgment mechanisms | reflection | “Certificates or reflection sessions motivate participation.” (S5) |

**Supplement 3. Expanded Theme Matrix**

| **Theme** | **Sub-Themes** | **Students (S)** | **Residents (R)** | **Faculty (F)** | **Interpretive Memo** |
| --- | --- | --- | --- | --- | --- |
| 1 Enhancing Medical Education through CBME | Early Exposure / Hands-On Learning | S4 “We know the theory…” | R3 “Exposure in second year…” | F2 “Students learn most when…” | Early contextual exposure bridges classroom and real-world learning. |
| 2 Addressing Health Disparities | Access & Trust | S7 “Patients from rural areas…” | R8 “People start trusting us.” | F5 “Polluted water and waste…” | Social and environmental inequities are interlinked; trust builds accountability. |
| 3 Challenges in Implementation | Logistics / Safety / Institutional Barriers | S2 “Female students cannot travel alone.” | R10 “No transport or funds.” | F4 “Without institutional support…” | Structural and safety barriers need institutional solutions. |
| 4 Integrating Environmental & Public Health | Pollution / Sustainability | S3 “Learning about pollution…” | R6 “Each module should mention…” | F1 “Green-clinic projects…” | Planetary health integration links education to sustainability. |
| 5 Strengthening Community Engagement | Partnerships / Mentorship | S6 “Following up…” | R9 “Community leaders help…” | F7 “Faculty mentorship sustains…” | Collaborations enhance continuity and trust. |
| 6 Monitoring & Evaluation | Reflection / Feedback | S5 “Certificates motivate…” | R12 “Collecting feedback…” | F6 “Tracking outcomes…” | Reflection and feedback ensure continuous quality improvement. |

# Supplement 4. CBME Integration Framework

## 4A. Year-by-Year Integration Map

| **Year** | **CBME Module / Rotation** | **Health Disparity Focus** | **Environmental Health Focus** | **Expected Competency Outcome** |
| --- | --- | --- | --- | --- |
| 1 | Community Orientation | Identify social determinants of health | Observe waste / sanitation issues | Describe community context & equity issues |
| 2 | Family Health Surveys | Equity in access to care | Water and air quality risks | Conduct household health assessments |
| 3 | Primary Care Rotation | Marginalised populations | Pollution & climate awareness | Plan community health promotion |
| 4 | Public Health Project | Health advocacy skills | Environmental policy analysis | Evaluate community interventions |
| 5 | Capstone CBME Elective | Integrated social & environmental responsibility | Sustainable practice design | Demonstrate social accountability |

4B. Sample Session Plan

| **Learning Outcome** | **Teaching Strategy** | **Resources** | **Assessment** |
| --- | --- | --- | --- |
| Analyse how poverty affects health behaviour | Problem-based discussion of local cases | Community data maps | Reflective essay rubric |
| Counsel families on environmental hazards | Field visit + role play | WHO guidelines, posters | Mini-CEX field counselling |

## **4C. Monitoring and Evaluation (M&E) Matrix**

(Logic Model for Integrating Health Disparities and Environmental Health into CBME)

| **Component** | **Description** | **Measurable Indicators / Means of Verification** |
| --- | --- | --- |
| **1 Inputs** | Institutional commitment & funding; trained faculty; community MOUs; teaching materials & transport; ethical approvals | Annual budget records; faculty training logs; signed partnership agreements; availability of transport & safety kits; IRB compliance reports |
| **2 Activities** | Orientation sessions; early & longitudinal placements (Y2–Y5); student-led health needs assessments & environmental audits; faculty supervision; community workshops | Attendance lists; field visit reports; photo documentation; supervision checklists; workshop registries |
| **3 Outputs** | Completed student projects; reflective reports & presentations; community awareness improvement; faculty development sessions | # projects per cohort; reflection rubric scores; community knowledge survey (pre/post); faculty feedback forms |
| **4 Short-Term Outcomes** | Enhanced student competence in equity & environmental awareness; increased faculty capacity; stronger university-community trust | Pre/post competency tests; faculty self-efficacy surveys; community satisfaction ≥ 80 % positive |
| **5 Intermediate Outcomes** | Integration of modules into core curriculum; institutional CBME policies; routine M&E reviews | Revised curriculum documents; number of policies endorsed; annual program review reports |
| **6 Long-Term Outcomes / Impact** | Improved community health indicators (hand-washing, waste segregation, clinic attendance); graduates show social accountability; replicable CBME model nationwide | Community surveys (2-yearly); graduate tracer studies; external HEC/PMDC accreditation reviews |

# Supplement 5. Sample Assessment Tools

### A. Reflective Journal Rubric (10 marks)

| **Criterion** | **Excellent (9–10)** | **Good (7–8)** | **Adequate (5–6)** | **Limited (≤4)** |
| --- | --- | --- | --- | --- |
| Critical Reflection | Integrates theory and practice deeply | Clear linkages with minor gaps | Mostly descriptive | Superficial |
| Self-Awareness | Shows growth and empathy | Some insight | Limited | None |
| Evidence / Examples | Specific community cases used | Some examples | Few examples | None |
| Organization | Logical and coherent | Mostly clear | Somewhat disorganized | Confusing |

B. Community Feedback Form

| **Dimension** | **Indicator** | **Scale (1 = Poor → 5 = Excellent)** |
| --- | --- | --- |
| Communication & Respect | Team listened and interacted politely | 1 2 3 4 5 |
| Usefulness of Visit | Visit helped address community health needs | 1 2 3 4 5 |
| Environmental Contribution | Promoted cleanliness / awareness | 1 2 3 4 5 |
| Overall Satisfaction |  | 1 2 3 4 5 |

C. Mini-CEX (Field Counseling Skill)

| **Skill Area** | **Competent** | **Needs Improvement** |
| --- | --- | --- |
| Builds rapport with community member | ☐ | ☐ |
| Explains disease / prevention clearly | ☐ | ☐ |
| Links advice to environmental context | ☐ | ☐ |
| Demonstrates empathy & professionalism | ☐ | ☐ |

D. Safety Checklist for Community Visits

| **Item** | **Checked (✓)** |
| --- | --- |
| Pre-visit risk assessment completed |  |
| Transportation arranged / insured |  |
| Supervisor contact shared |  |
| Emergency protocol briefed |  |
| PPE / First Aid available |  |
