# Supplementary material for: Integrating health disparities and environmental health into community-based medical education: a qualitative study
Source: BMC Med Educ. 2026 Jan 27;26:311. doi: 10.1186/s12909-025-08485-w (PMC12918272; doi:10.1186/s12909-025-08485-w)
Supplement: Supplementary file 2 — Supplementary Material 2. [file 12909_2025_8485_MOESM2_ESM.docx]

**Annexure 1**

1. Can you briefly describe your experiences with community-based medical education so far?
2. How are health disparities *or* social determinants of health addressed during community placements?
3. Have environmental or public health topics (e.g., pollution, sanitation, sustainability) been included?
4. What learning activities (field visits, projects, reflections) helped you engage with community health issues?
5. How relevant or effective do you find current CBME teaching for understanding real-world community health needs?
6. What challenges or barriers have you observed in teaching or learning about health disparities or environmental/public health?
7. What institutional or logistical factors influence the success of community-based learning?
8. How can CBME better integrate equity, environmental awareness, and sustainability?
9. What role should students, residents, faculty, and community members play in improving CBME?
10. What strategies or supports would make such integration more feasible and sustainable?
11. How could reflection, feedback, or evaluation be improved within CBME activities?
12. Do you have any final suggestions for strengthening CBME in your institution?
